# Supplementary material for: Discovery of Novel 1,2,3-triazole Derivatives as IDO1 Inhibitors
Source: Pharmaceuticals (Basel). 2022 Oct 25;15(11):1316. doi: 10.3390/ph15111316 (PMC9695734; doi:10.3390/ph15111316)

Figure S1. ^{1}H NMR spectrum (600MHz, DMSO-d_6) of compound 3a

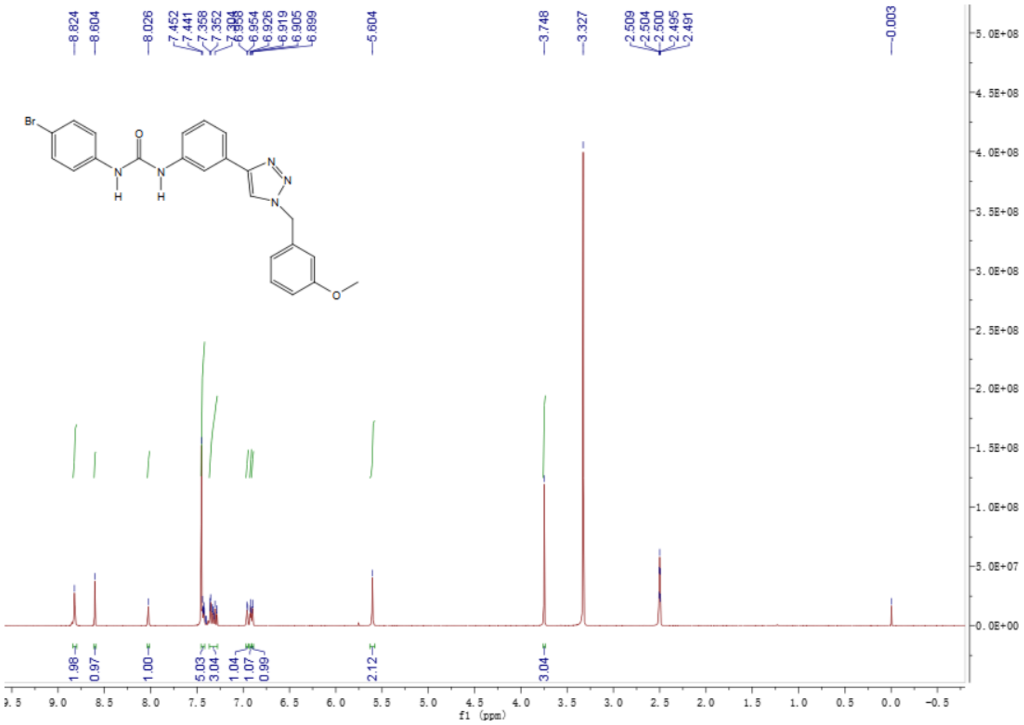

Figure S2. ^{1}H **NMR spectrum (600MHz, DMSO-d$_6$) of compound 3b**

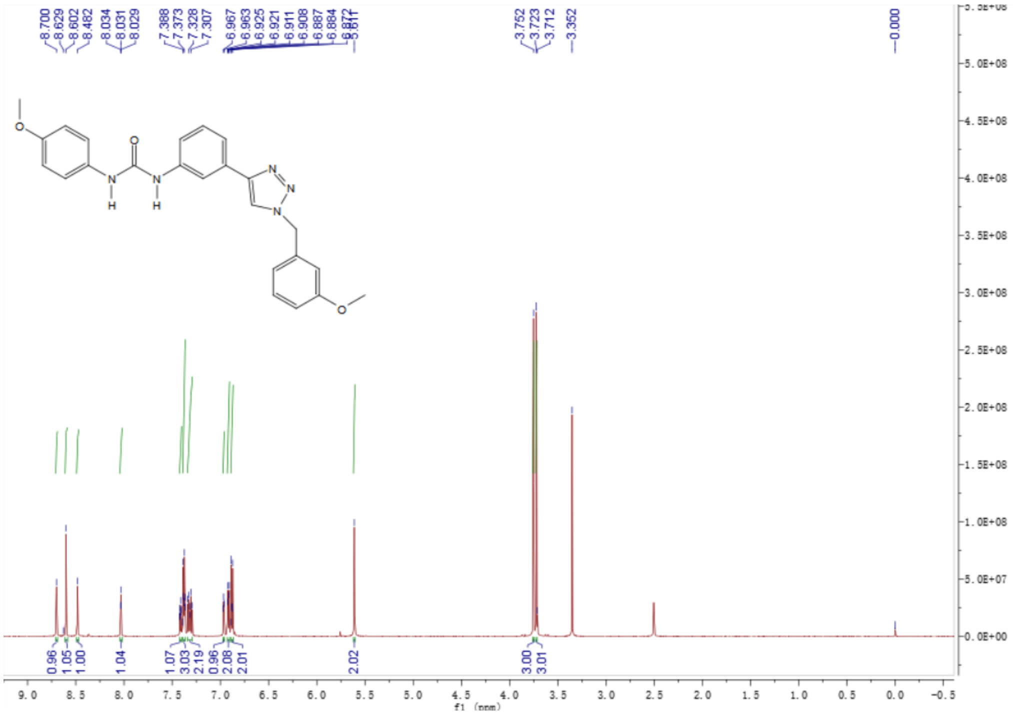

Figure S3. ^{1}H NMR spectrum (600MHz, DMSO-d$_6$) of compound 3c

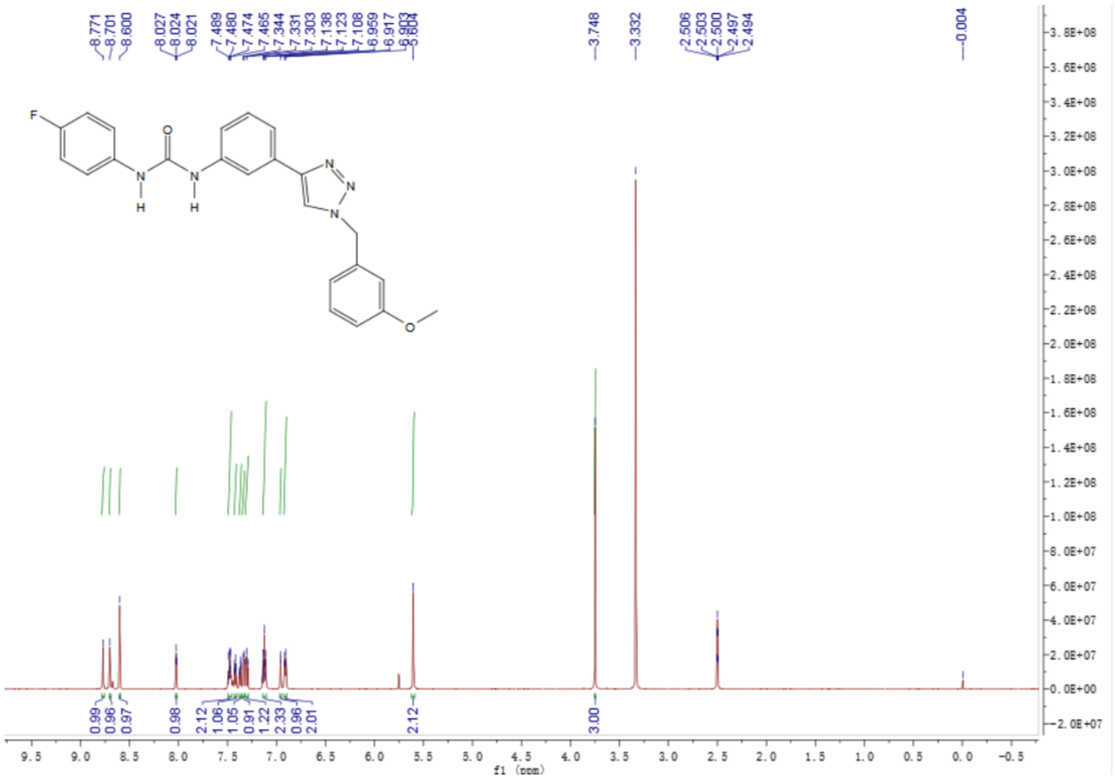

Figure S4. ^{1}H NMR spectrum (600MHz, DMSO-d_6) of compound 3d

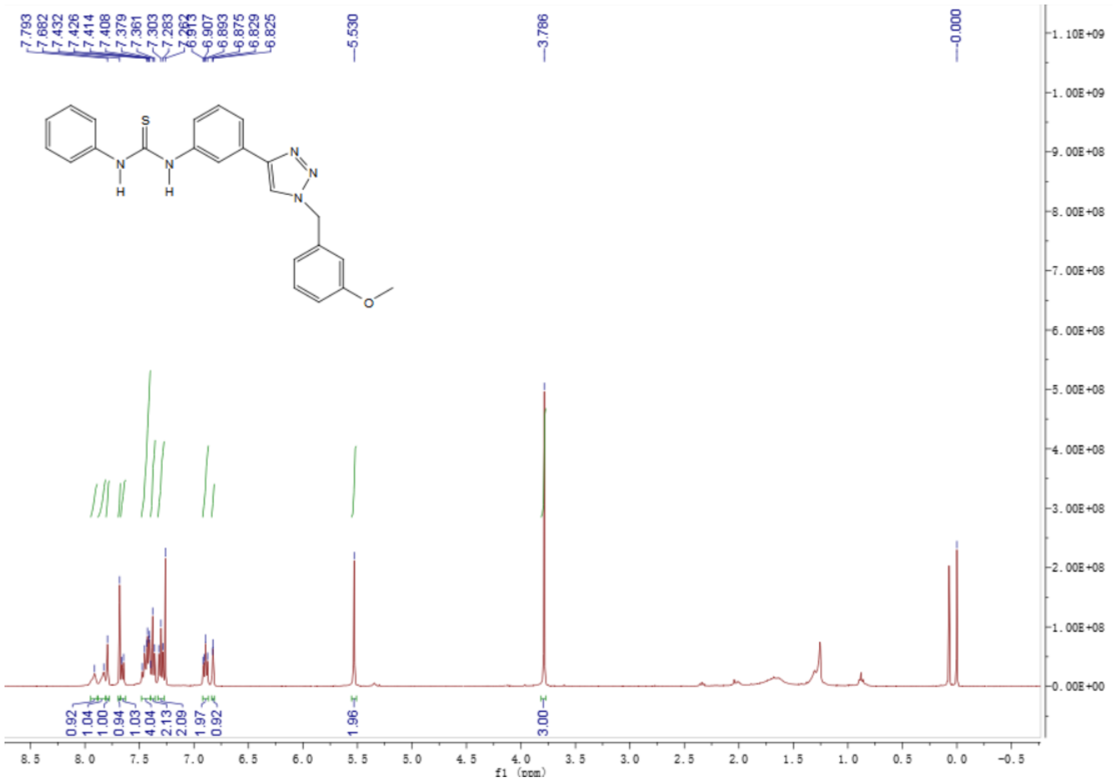

Figure S5. ^{1}H NMR spectrum (600MHz, DMSO-d_6) of compound 3e

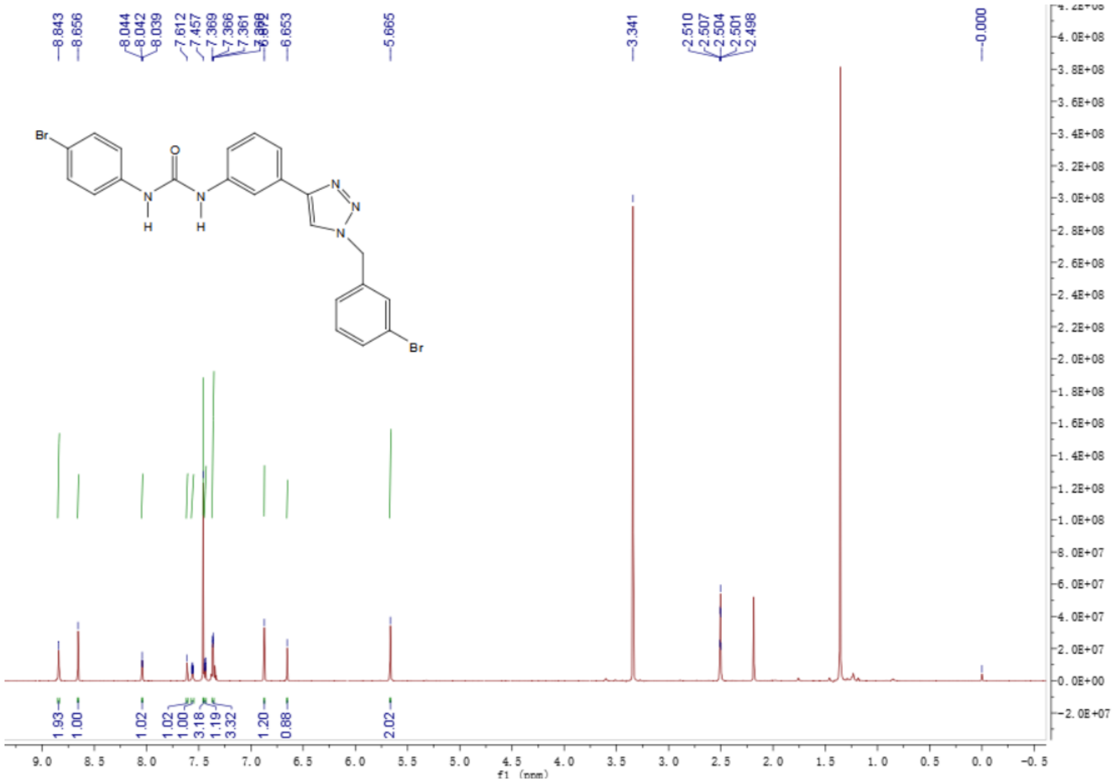

Figure S6. ^{1}**H NMR spectrum (600MHz, DMSO-d$_6$) of compound 3f**

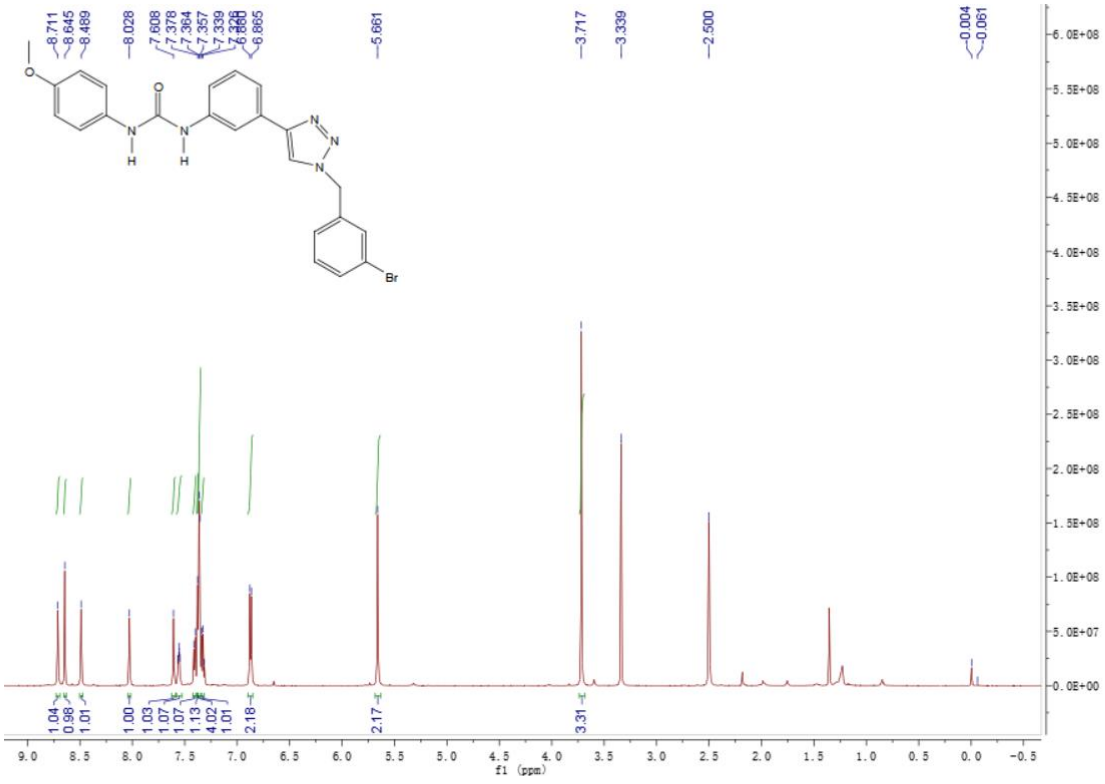

Figure S7. ^{1}H NMR spectrum (600MHz, DMSO-d_6) of compound 3g

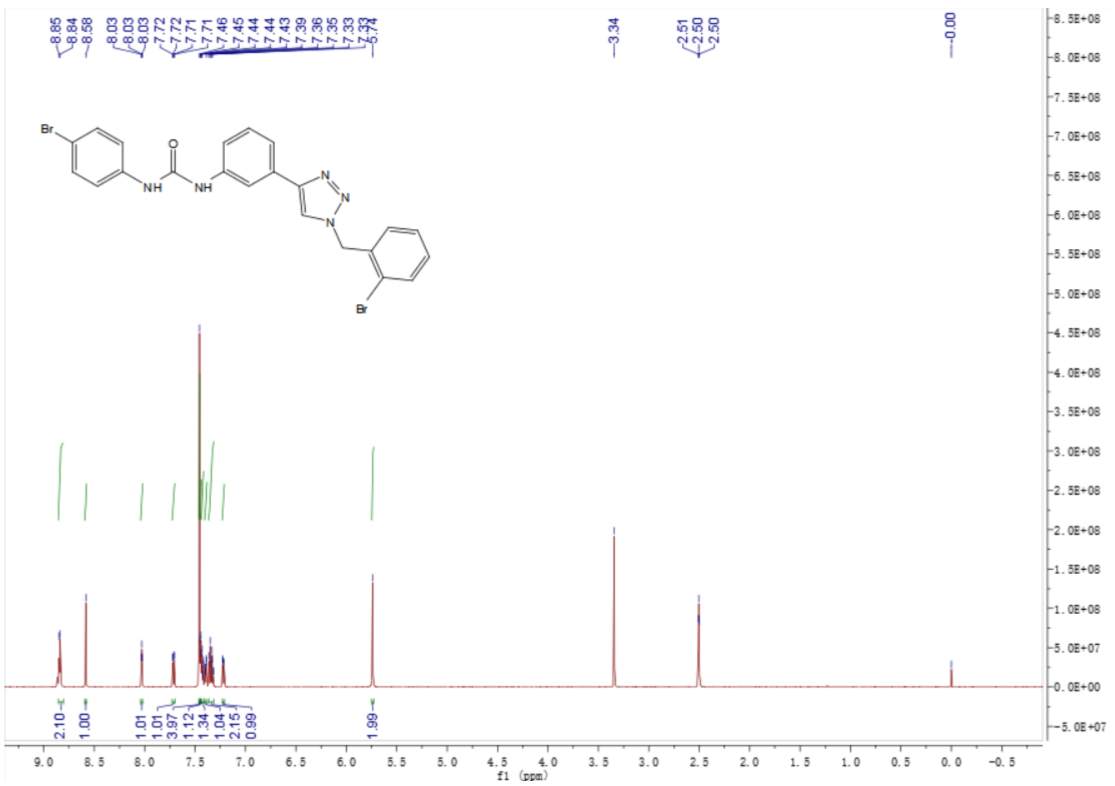

Figure S8. ^{1}H NMR spectrum (600MHz, DMSO-d6) of compound 3h

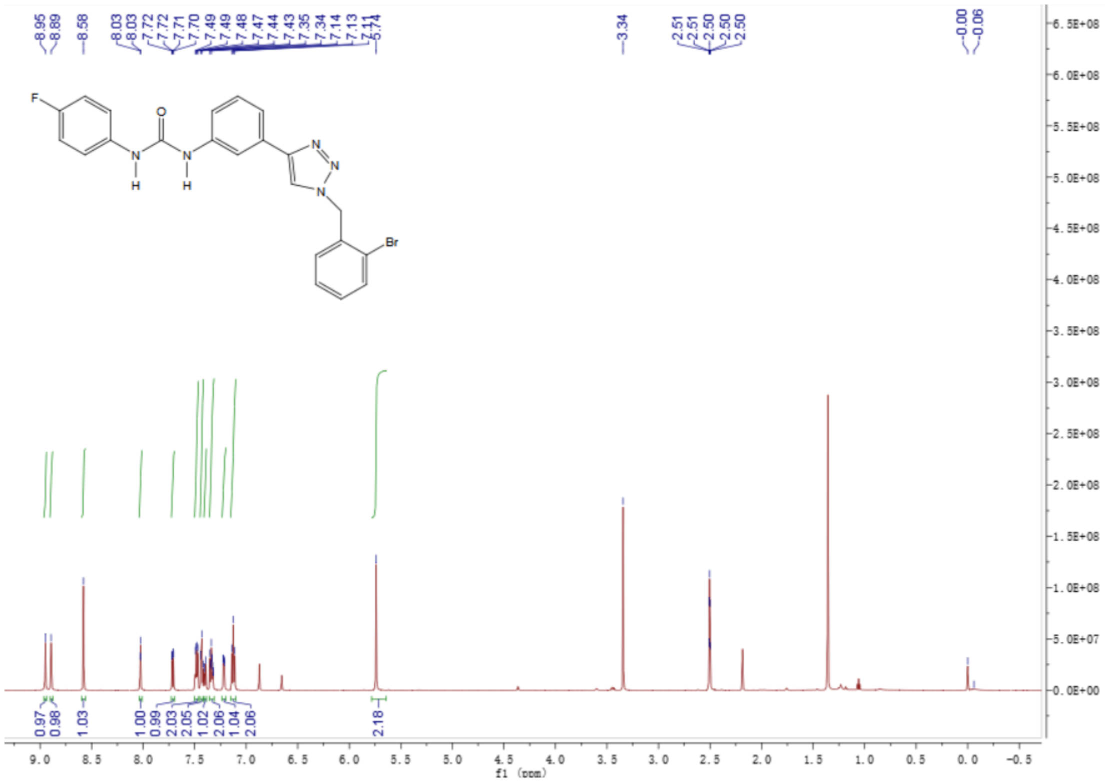

Figure S9. ^{1}H NMR spectrum (600MHz, DMSO-d_6) of compound 3i

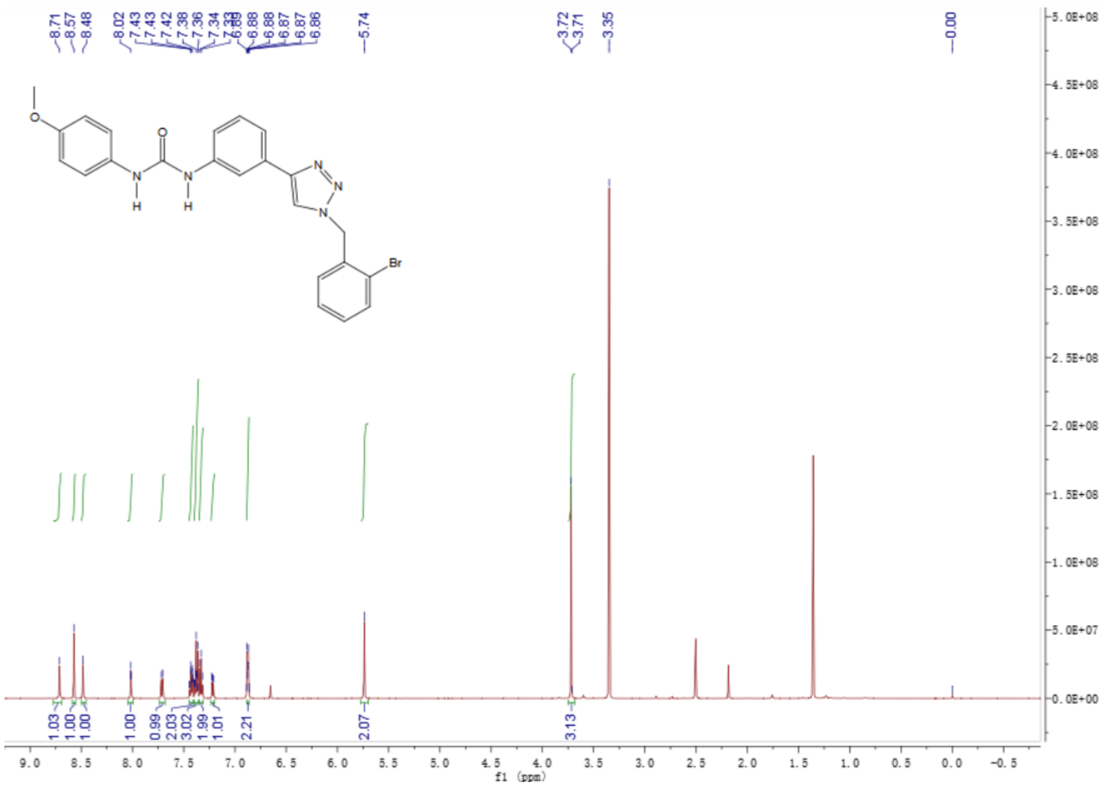

Figure S10. ^{1}H NMR spectrum (600MHz, DMSO-d$_6$) of compound 5a

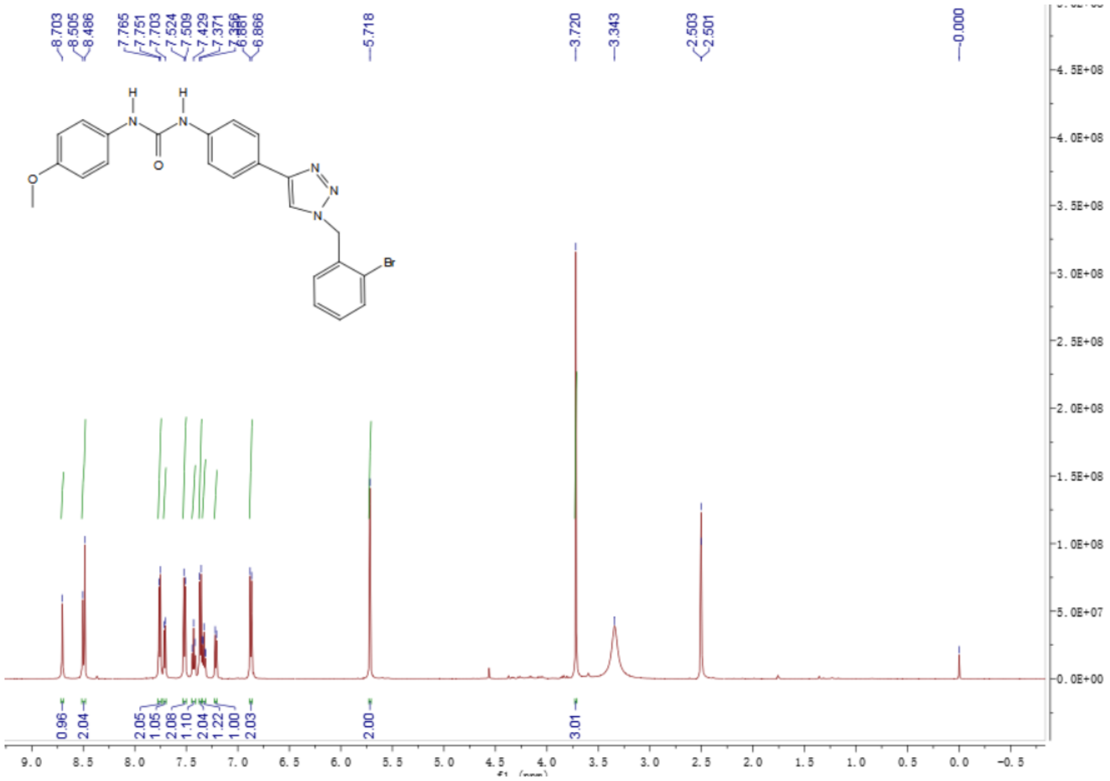

Figure S11. ^{1}H NMR spectrum (600MHz, DMSO-d_6) of compound 5b

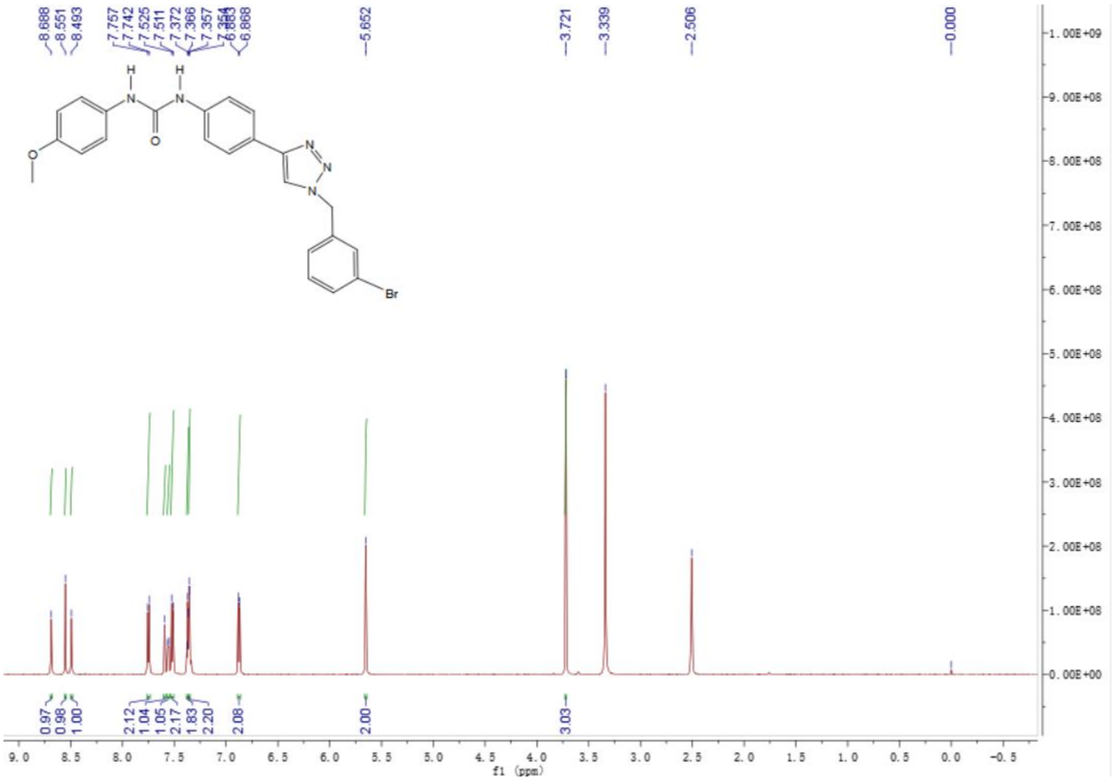

Figure S12. ^{1}H NMR spectrum (600MHz, DMSO-d6) of compound 5c

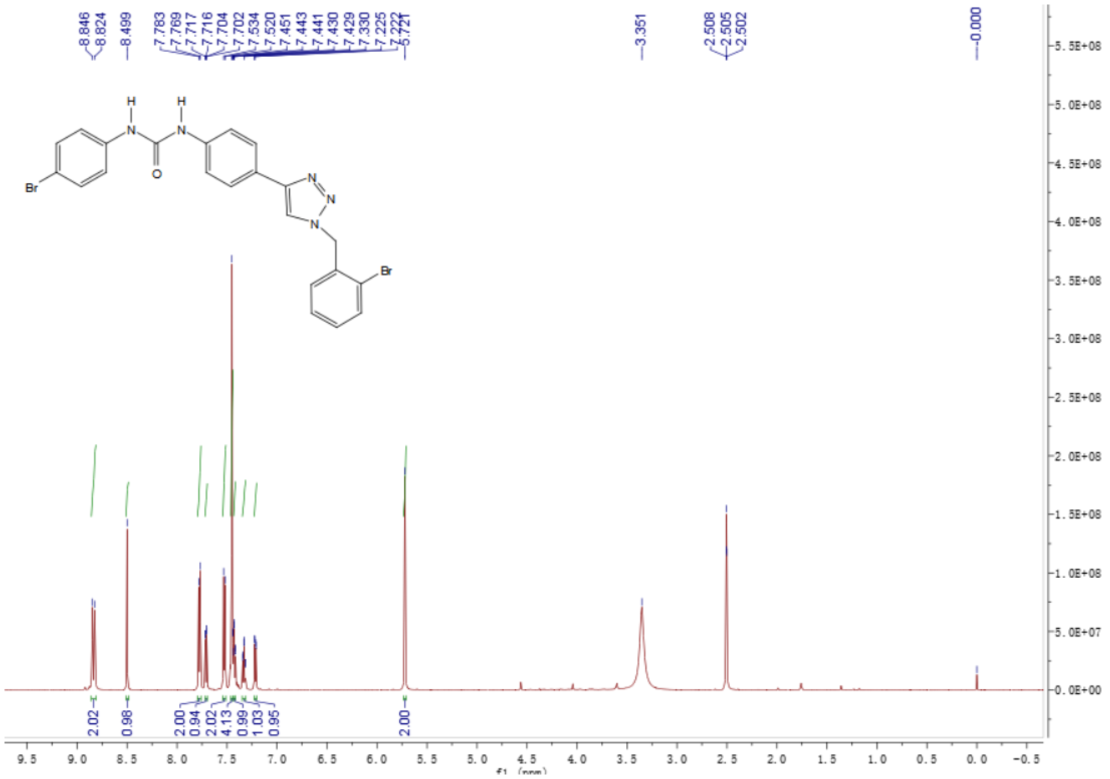

Figure S13. ^{1}H NMR spectrum (600MHz, DMSO-d_6) of compound 5d

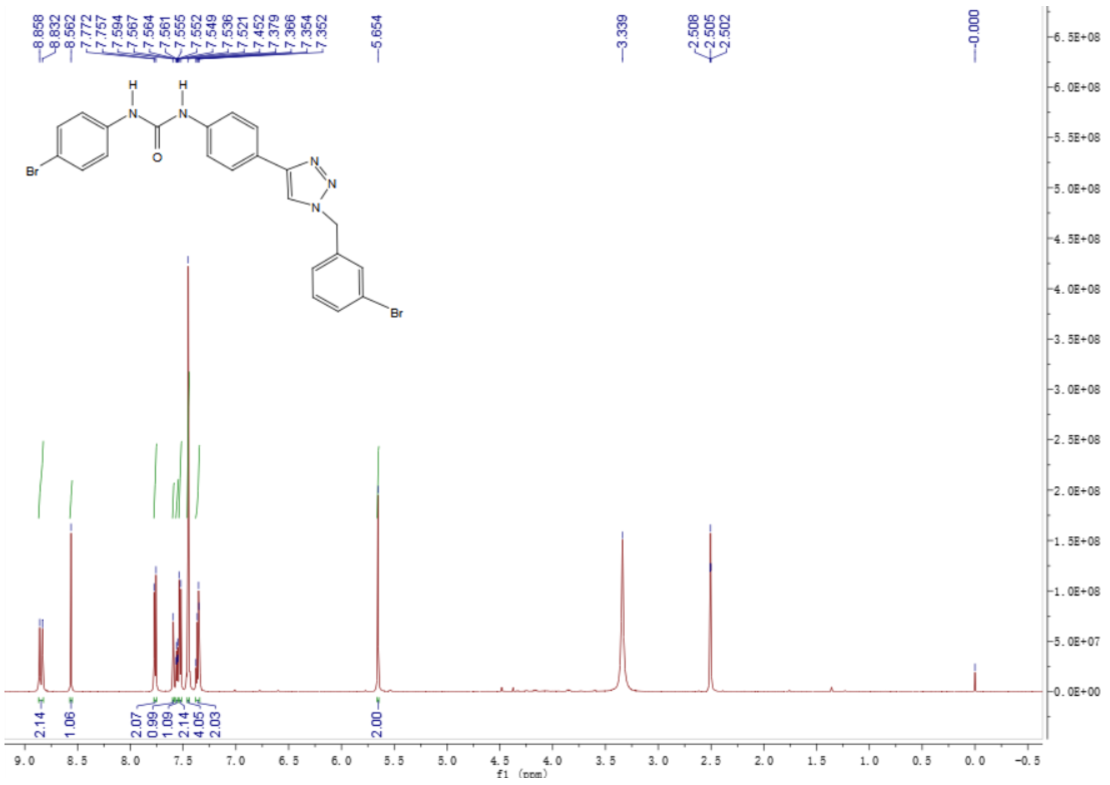

Figure S14. ^{1}H NMR spectrum (600MHz, DMSO-d_6) of compound 5e

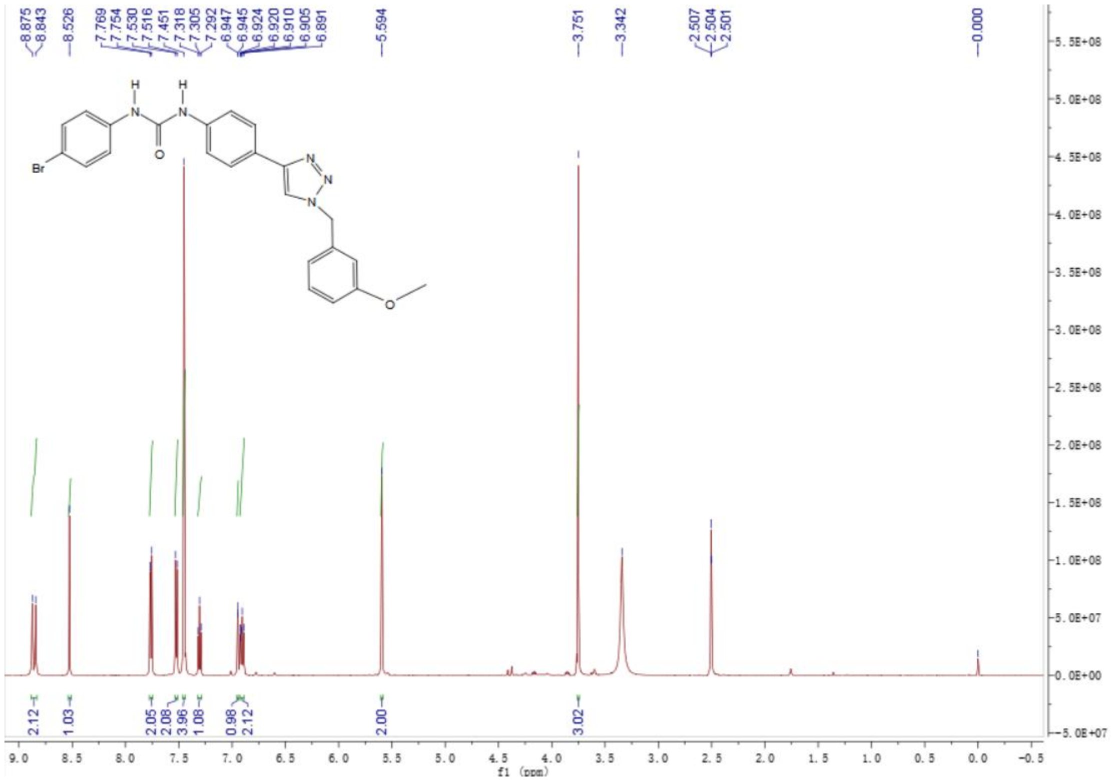

Figure S15. ^{1}H NMR spectrum (600MHz, DMSO-d6) of compound 5f

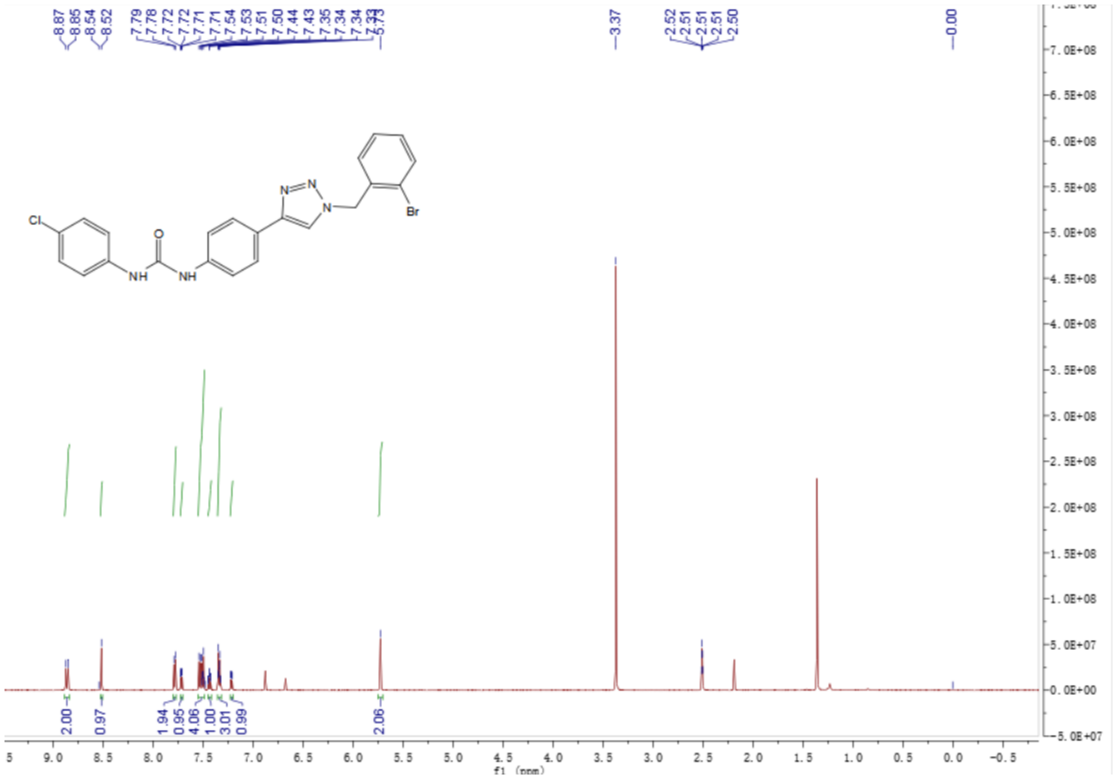

Figure S16. ^{1}H NMR spectrum (400MHz, DMSO-d_6) of compound 7a

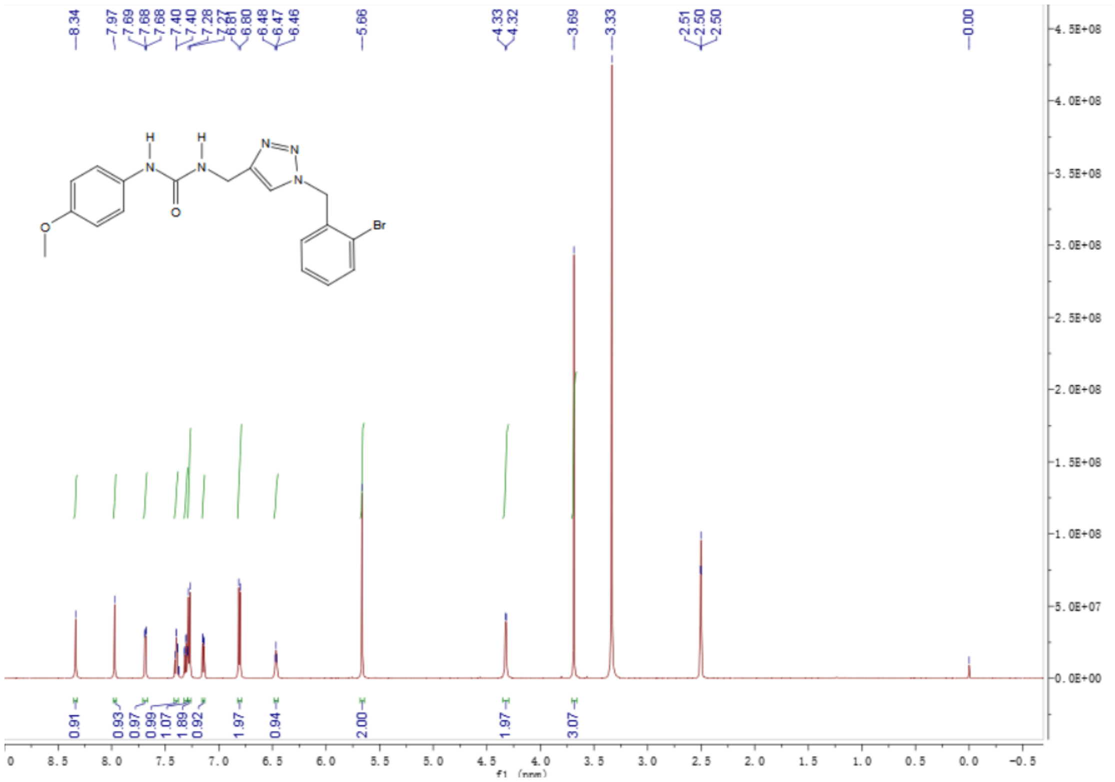

Figure S17. ^{1}H NMR spectrum (400MHz, DMSO-d$_6$) of compound 7b

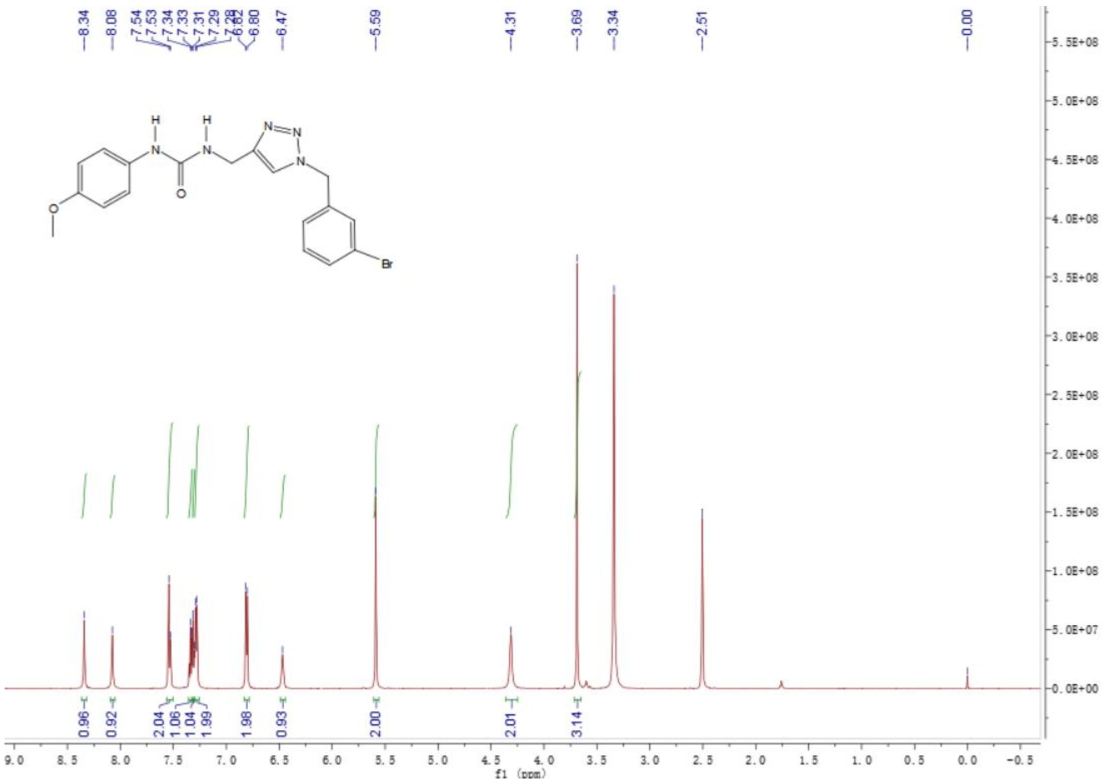

Figure S18. ^{1}H NMR spectrum (400MHz, DMSO-d_6) of compound 7c

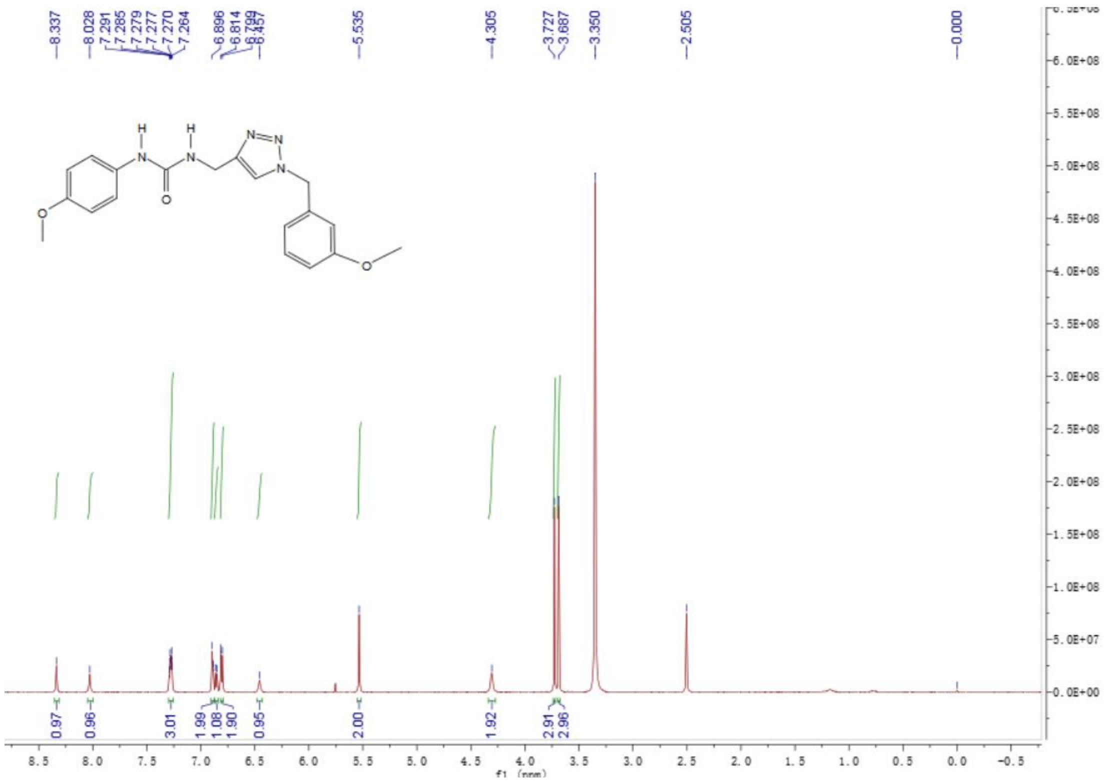

Figure S19. ^{1}H NMR spectrum (400MHz, DMSO-d_6) of compound 7d

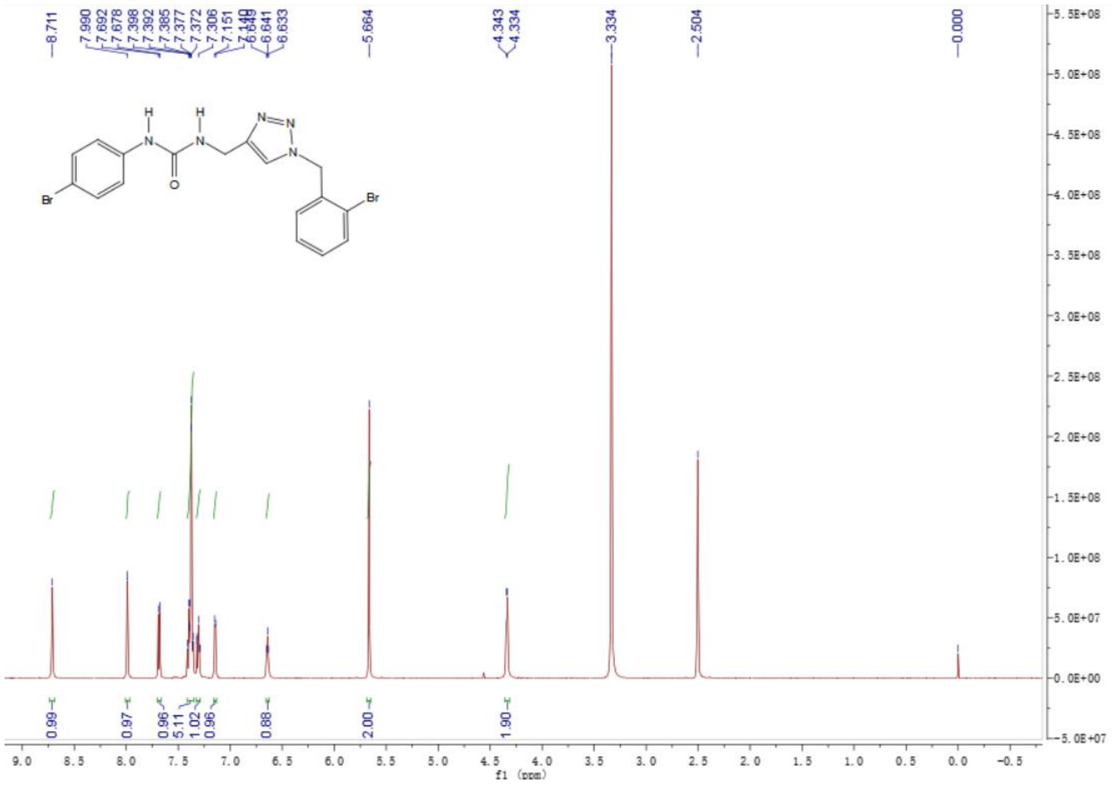

Figure S20. ^{1}H NMR spectrum (400MHz, DMSO-d_6) of compound 7e

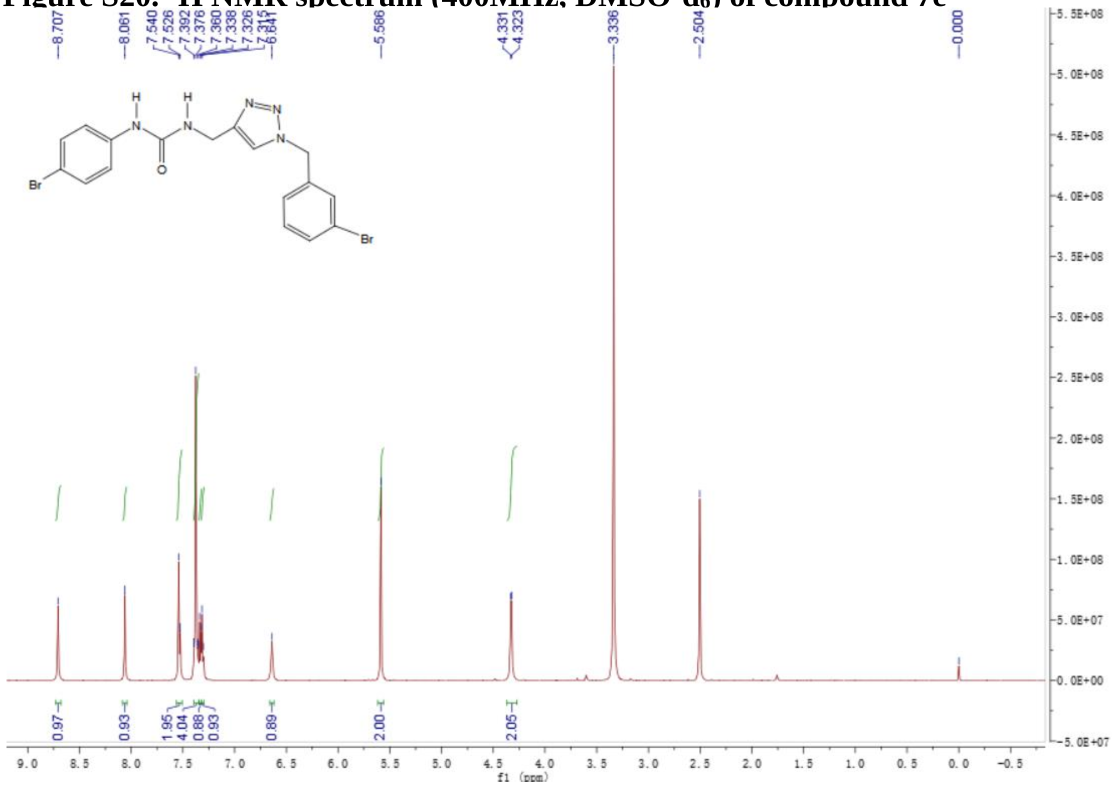

Figure S21. ^{1}H NMR spectrum (400MHz, DMSO-d$_6$) of compound 7f

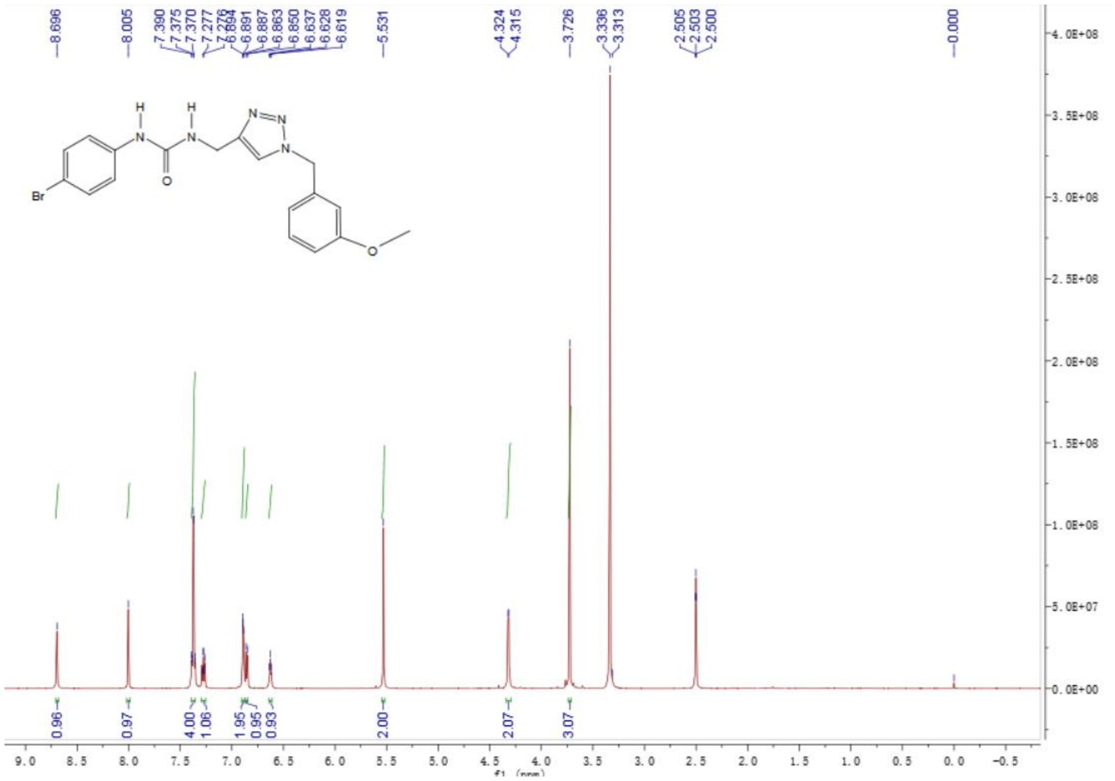

Supplement: Supplementary file 1 [file pharmaceuticals-15-01316-s001.zip › pharmaceuticals-1978433-Supplementary Information.pdf]
